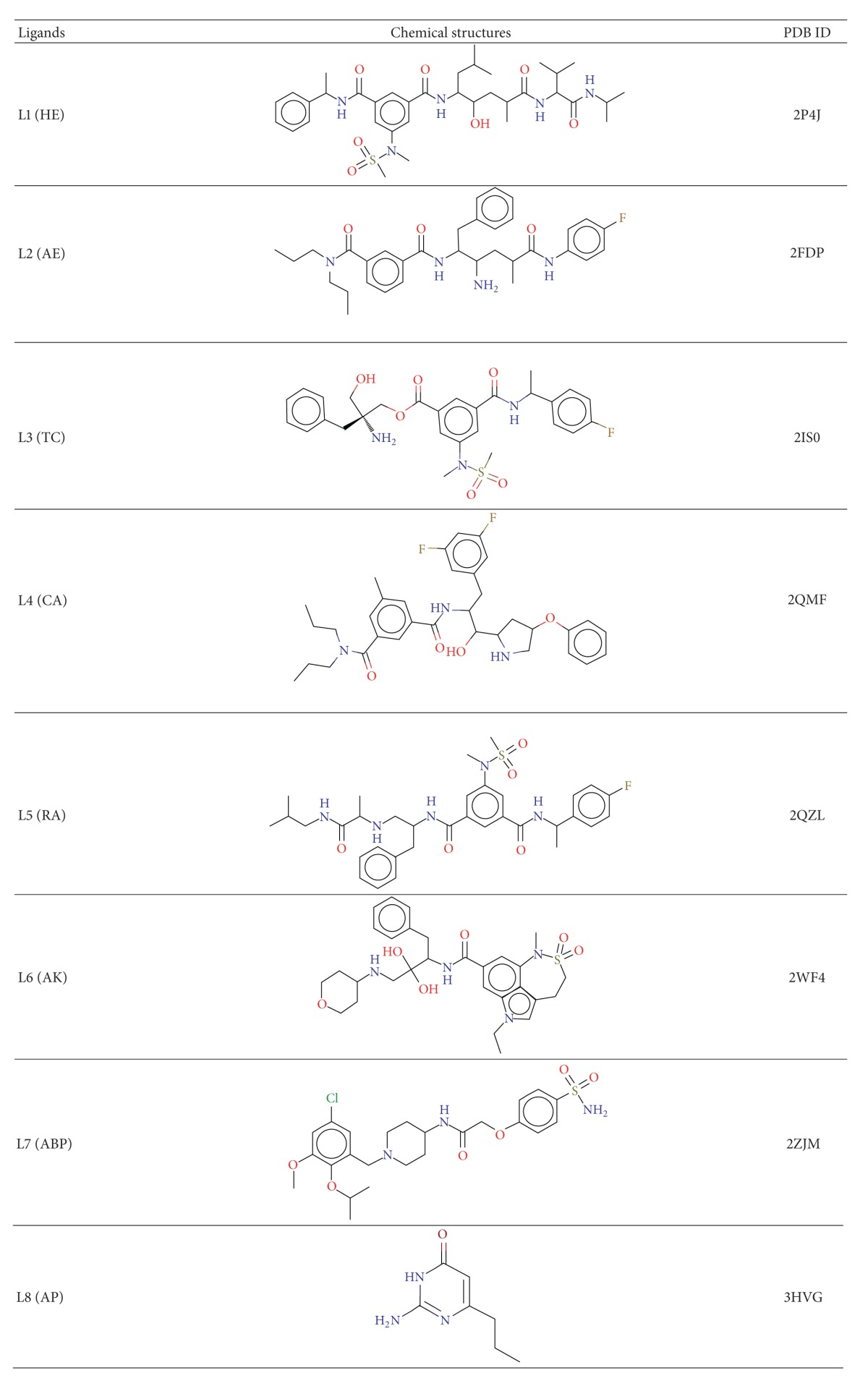# Computational Insights into Substrate and Site Specificities, Catalytic Mechanism, and Protonation States of the Catalytic Asp Dyad of ***β***-Secretase

**DOI:** 10.1155/2014/598728

**Published:** 2014-09-18

**Authors:** Arghya Barman, Rajeev Prabhakar

**Affiliations:** Department of Chemistry, University of Miami, 1301 Memorial Drive, Coral Gables, FL 33146, USA

## Abstract

In this review, information regarding substrate and site specificities, catalytic mechanism, and protonation states of the catalytic Asp dyad of *β*-secretase (BACE1) derived from computational studies has been discussed. BACE1 catalyzes the rate-limiting step in the generation of Alzheimer amyloid beta peptide through the proteolytic cleavage of the amyloid precursor protein. Due to its biological functioning, this enzyme has been considered as one of the most important targets for finding the cure for Alzheimer's disease. Molecular dynamics (MD) simulations suggested that structural differences in the key regions (inserts A, D, and F and the 10s loop) of the enzyme are responsible for the observed difference in its activities towards the WT- and SW-substrates. The modifications in the flap, third strand, and insert F regions were found to be involved in the alteration in the site specificity of the glycosylphosphatidylinositol bound form of BACE1. Our QM and QM/MM calculations suggested that BACE1 hydrolyzed the SW-substrate more efficiently than the WT-substrate and that cleavage of the peptide bond occurred in the rate-determining step. The results from molecular docking studies showed that the information concerning a single protonation state of the Asp dyad is not enough to run an in silico screening campaign.

## 1. Introduction

Alzheimer's disease (AD) is a progressive neurological disorder and common cause of dementia, which affects 4–8% of the elderly population worldwide. Scientific efforts to slow the progression of AD could save society $ 1.2 trillion to $ 3.97 trillion a year by 2050 in cost. The formation of extracellular amyloid (or senile) plaques and intracellular neurofibrillary tangles at the hippocampus and cortical grey regions of the brain are considered to be the pathological hallmark for this life threatening disease. It has also been postulated that plaques are not neurotoxic and more soluble; partly aggregated complexes are the main cause of this disease. These amyloid plaques are developed due to the aggregation and deposition of 1–40/42 amino acid containing small peptides known as amyloid beta (A*β*) peptide(s) [1, 2]. Inside the brain, A*β* peptide(s) are produced by the sequential cleavage of the membrane bound amyloid precursor protein (APP) by two extremely critical enzymes called *β*-secretase (BACE1) and *γ*-secretase.

BACE1 belongs to a ubiquitous family of aspartyl proteases [3–6] which catalyzes the hydrolytic cleavage of a large number (~80) of proteins that play critical roles in several cellular and subcellular pathways [7, 8]. BACE1 cleaves the Met671-Asp672 amide bond of amyloid precursor protein (APP) at the extracellular space and initiates the formation of A*β* peptide(s) [9–11]. In addition, BACE1 can cleave a double mutant (Lys670 → Asn and Met671 → Leu) of APP known as the Swedish mutant (SW) with sixty times higher efficiency than the WT-substrate [12]. Since BACE1 plays a critical role in the rate-limiting steps of A*β* production, the inhibition of this enzyme became widely acknowledged to regulate the production of A*β* peptide(s) [13]. Based on a wealth of experimental data including genetics, biochemical studies, and animal modeling this enzyme has been proposed to be a very promising target for the treatment of AD [9–14].

Structurally, the ectodomain of BACE1 is composed of several subregions that control the entry and orientation of the substrate at the active site. The active site of BACE1 contains two conserved Asp residues [15] that forms the catalytic dyad. This dyad has been implicated in the catalytic functioning of the entire family of aspartyl proteases including pepsin, renin, cathepsin D, and HIV protease [16–23]. The catalytic Asp dyad at the active site is covered over by an antiparallel hairpin-loop known as flap. The mechanisms of flap closing and catalysis are of great significance due to their involvement in human diseases such as AD. During the catalytic cycle, the flap must open to allow the entrance of the substrate into the active site cleft and steer it towards the catalytic Asp dyad to attain a reactive conformation. In this conformation, the specific peptide bond(s) of the substrate is hydrolytically cleaved. This type of gating mechanism has been reported to be utilized by most of the members of the aspartyl protease family. The dyad utilizes a general acid-base mechanism for the catalysis of peptide hydrolysis [17]. Although, theoretical, X-ray, and neutron diffraction data show that the Asp residues in the catalytic dyad can switch protonation states during the catalytic turnover [24–26], the effect of the protonation states of these residues still remains an intriguing issue in the development of successful drug designing strategies.

Although experimental techniques can provide a great deal of information about the catalytic mechanism and protonation states of the ionizable residues, the atomic level description of these complex chemical transformations is still beyond their reach. These limitations can be overcome by modern computational chemistry methods that can describe these complex processes at the atomistic level. Computational chemistry is a well-established field which has become an indispensable tool to study complex chemical and biochemical systems. In the last decade, applications of the density functional theory (DFT) to study the chemical reactions using small systems have dominated the field. However, the main caveat of using DFT is the restricted number of atoms (~200). Therefore, to study the larger system, DFT (QM) has been coupled to the molecular mechanics (MM) potentials and implemented in hybrid QM/MM(ONIOM) [27–33] method to study the catalytic mechanisms of the enzymes. Applications of QM/MM in biological systems have shown tremendous success [34]. On the other hand, molecular dynamics (MD) simulations have become an essential part of current research to study the phase space behavior, conformational changes of molecules, and to calculate thermodynamic properties of systems [35, 36]. Along with MD, MM based scoring functions have also been incorporated into the docking engines and have gained extensive use in modern computer aided drug design protocol [37].

In this review, the current knowledge of structural and functional aspects of BACE1 in atomistic detail using multidimensional computational methods has been discussed.

## 2. Structural Characteristics of BACE1

**Figure 1:**
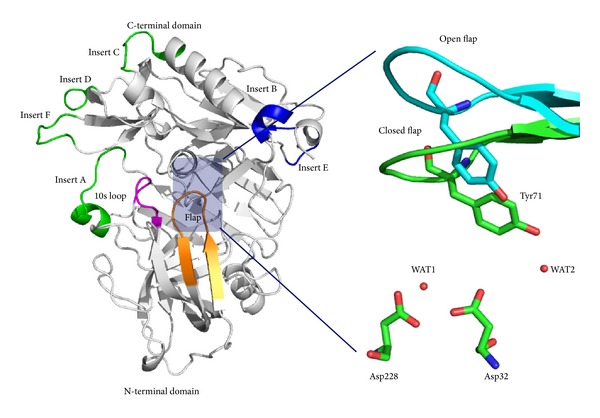
Critical regions of BACE1 that participate in the substrate recognition, catalytic Asp dyad, conserved waters (WAT1 and WAT2), and orientation of Tyr71 in open and closed conformation of the flap.

To date, more than 290 crystal structures of BACE1 (Apo form and cocrystal with inhibitors) have been deposited into the protein data bank (PDB). However, the first cocrystal structure of BACE1 with a hydroxyethylene (HE) based transition state isostere (OM99-2 and OM00-3) revealed the first evidence of BACE1 active site that contained the catalytic dyad (Asp32 and Asp228) at the center of the active site [15, 38]. The globular nature of BACE1 can be divided into N- and C-terminal domains. The flap of this enzyme is composed of eleven residues (Val67-Glu77) and is positioned at the N-terminal domain. A conserved Tyr71 residue is located at the flap which is found to adopt different conformations in the presence and absence of inhibitors (Figure 1). There are several key functional regions, namely, 10s loop (Lys9-Tyr14), third strand (Lys107-Gly117), and insert A (Gly158-Leu167) that are present at the N-terminus. Whereas insert B (Lys218-Asn221), insert C (Ala251-Pro258), insert D (Trp270-Thr274), insert E (Glu290-Ser295), and insert F (Asp311-Asp317) regions are located at the C-terminus, these regions facilitate the entry and binding of different substrates at the active site through their movements [15, 39]. At the active site, BACE1 contains two conserved water molecules (WAT1 and WAT2). After carefully analyzing 82 cocrystal structures of aspartyl proteases, the specific role of these two water molecules was suggested by Andreeva and Rumsh [40]. The WAT1 water located near the catalytic Asp dyad was assigned to be most important as it is utilized in the hydrolytic cleavage of the peptide bond. The second water molecule (WAT2) participates in a continuous H-bonding network and stabilizes the flap in the closed conformation through structural organization.

## 3. Differential Substrate Specificity of BACE1

The induced fit model for enzyme-substrate interactions [41] suggests that the interaction of the substrate with an enzyme modulates the spatial orientation of the enzyme's active site residues into an optimal and precise conformation to accommodate the substrate. Conformational preference of the enzyme-substrate complex is driven by the dynamics of both the enzyme and substrate. It is not possible to resolve X-ray structure of a wild type- (WT-) enzyme in active form bound to substrate. In the absence of this structure, the atomistic level understanding of enzyme-substrate interactions remains elusive. We have studied the role of protein dynamics in the formation of enzyme-substrate complex and substrate specificity of BACE1 involved in the generation of A*β*-peptides [23].

**Figure 2:**
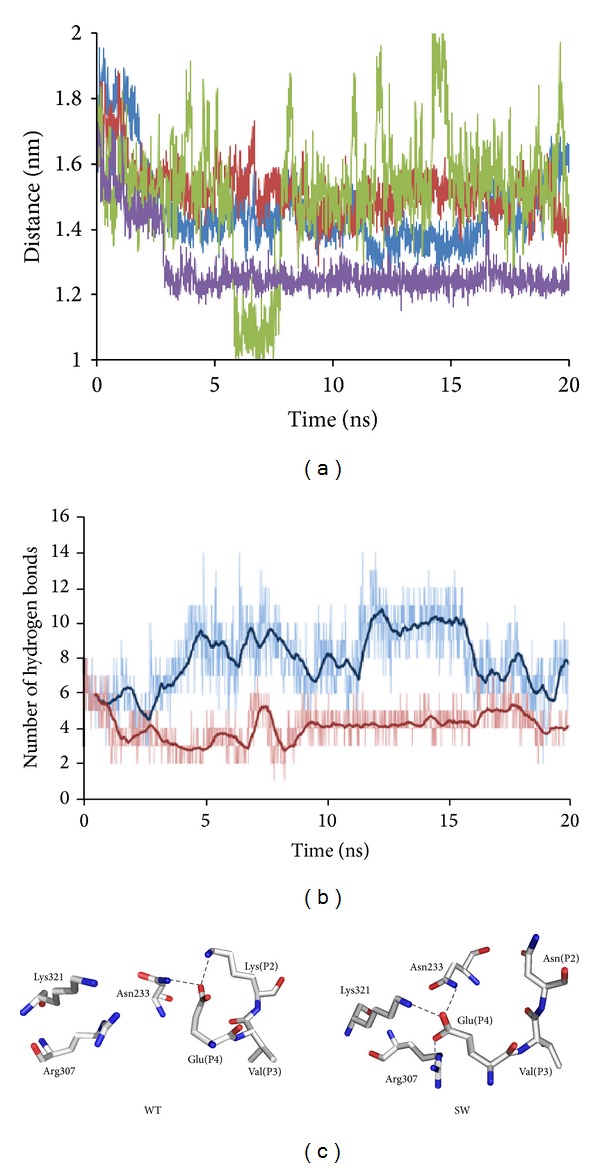
(a) Time evolution of the topological distance between the C^α^(Thr72) of flap tip and C^*β*^(Asp32) of catalytic dyad (violet: BACE1-compound 11, blue: BACE1-SW substrate, red: BACE1-WT substrate, and green: Apo BACE1). (b) Number of hydrogen bonds formed between the substrates and BACE1 (blue: BACE1-SW substrate, red: BACE1-WT substrate). (c) Interaction of the substrate Glu with the Arg307 of BACE1.

BACE1 is known to cleave the SW-substrate (Glu-Val-Asn-*Leu-Asp*-Ala-Glu-Phe) sixty times more efficiently than the WT-substrate (Glu-Val-Lys-*Met-Asp*-Ala-Glu-Phe). We explored the interaction profiles of BACE1 with the WT- and SW-substrates through 20 ns all-atom MD simulations in a TIP4P explicit water system using the OPLS-AA [42] force field. Four different structures (WT-BACE1, SW-BACE1, and a 4-phenoxypyrrolidine-based BACE-1 inhibitor (compound 11, PDB ID: 2qmg [43]) bound BACE1) [44] along with the apo form of BACE1 were studied. In order to capture the dynamic behavior of substrate recognition by the enzyme, the starting models were generated by placing the substrates and the inhibitor inside the apo form of BACE1 (PDB ID:1w50) [15, 45]. The representative structure derived from the compound 11-BACE1 simulations shows an accurate reproduction of the crystallographic orientation of the flap, the 10s loop, and BACE1- inhibitor interactions. The root-mean-square (rms) deviation between the simulated and X-ray structures was only 1.3 Å. The simulation therefore served as a validation of the MD method and the parameters that were used for the substrate bound BACE1. In the presence of the inhibitor and the substrate, the flap closing event (Figure 2) occurred around 3 ns and two water molecules facilitated the process. According to a site-directed mutagenesis study, electrostatic interactions between the Glu residue of the substrate and Arg307 residue of BACE1 play important roles in enhancing the catalytic efficiency of the enzyme [46]. In the BACE1-WT simulation, this interaction was lost due to the formation of intramolecular salt-bridge between the Glu residue and the Lys residue of the substrate (Figure 2). Therefore, the loss of the Glu-Arg307 interaction was considered as one of the causes for the diminished activity of the enzyme with the WT-substrate. A comparison between three critical topological distances (C^α^(Thr72)-C^*β*^(Asp32), C^α^(Thr72)-C^α^(Thr329), and OG1(Thr72)-NH1(Arg235)) and the volume of the active site showed that, in comparison to the WT-substrate, the flap was more closed and the active site was more constricted upon the binding of the SW-substrate. In addition, the number of hydrogen bonds formed by the SW-substrate was found to be two times (8–10) more than the bonds contributed by the WT-substrate. The computed electrostatic binding energy of the SW-substrate was found to be 1.9 kcal/mol more favorable than the WT-substrate. In addition to substrate-enzyme interactions, structures of inserts A, D, and F and the 10s loop of the enzyme were also substantially different upon the binding of these two substrates. Overall, these structural differences between the WT- and SW-substrate explicitly indicate that BACE1 demonstrated greater affinity for the SW-substrate and positioned it in a more bioactive conformation compared to the WT-substrate.

## 4. Differential Site Specificity of BACE1

In addition to the cleavage of Met-Asp amide bond of WT-APP at +1 site or *β* site, BACE1 can cleave the peptide bond between Tyr-Glu at +11 or *β*′ site and produce +11 A*β* species. This site specificity can be altered through the modification of C-terminal domain of BACE1. Glycosylphosphatidylinositol (GPI) bound form of BACE1 (BACE1-GPI chimera) was shown to have more *β* site cleavage specificity compared to the *β*′ site. Our MD simulation studies on BACE1 and BACE1-GPI showed that GPI could introduce significant changes in the structure of BACE1. The structural changes are associated with the flap, third strand, and insert F of the enzyme. In the presence of GPI, the flap moved to a more open conformation and strong hydrogen bond interactions between the third strand and insert F brought them closer to each other. On the other hand, the flap remains in a relatively closed conformation and the two segments moved apart due to the loss of the hydrogen bond interactions in the WT-BACE1. Overall, these results suggest that GPI induces structural modifications that may regulate the site recognition of BACE1 [47].

## 5. Catalytic Mechanism of BACE1 

BACE1 cleaves specific peptide bonds of the substrates utilizing the catalytic Asp dyad. This Asp dyad interacts with the conserved water molecule (WAT1) present in the active site and utilizes it for peptide hydrolysis. Recent X-ray and neutron diffraction data and theoretical calculations on the aspartyl proteases show that during the catalytic cycle, one of the Asp residues is protonated and the second one is unprotonated [24–26, 48]. BACE1 catalyzes the hydrolytic cleavage of the Met-Asp and Leu-Asp peptide bonds of the WT- and SW-substrate, respectively.

**Figure 3:**
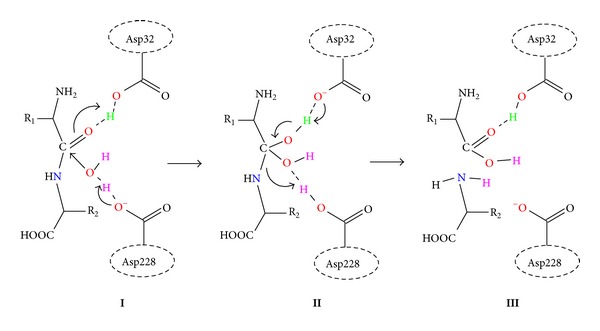
General acid-base mechanism utilized for peptide hydrolysis.

In the catalytic cycle, BACE1 utilizes a two-step general acid/base mechanism to cleave these peptide bonds (Figure 3) [17]. In the first step, from the reactant (**I**) the unprotonated Asp228 functions as a base and abstracts a proton from the catalytic water (WAT1) to generate a hydroxyl ion (OH^−^), which subsequently makes a nucleophilic attack on the carbonyl carbon of the peptide bond. In this process, Asp32 acts as an acid and concomitantly donates its proton to the carbonyl oxygen atom of the scissile peptide bond to generate the gem-diol intermediate (**II**). In** II**, two hydroxyl groups are coordinated to the carbonyl carbon atom of the peptide bond. In the next step, the two aspartate residues exchange their functional roles. Asp32 now functions as a base and abstracts a proton from the hydroxyl group (–OH) of** II**. Here, Asp228, which initially acted as a base and became protonated in the previous step, plays the role of an acid by donating its proton to the amide nitrogen atom (–NH) of the scissile peptide bond. The cleavage process of the peptide bond occurs in this process and generates separated amine (–NH_2_) and carboxyl (–COOH) terminals (**III**).

The hydrolysis of both WT- and SW-substrates was investigated using two computational methods. In the first step of this study, the most representative structures derived from the BACE1-WT and BACE1-SW MD simulations were utilized to develop pruned models of the enzyme-substrate complexes. In the second step, these structures were used to investigate hydrolytic cleavage mechanisms through density functional theory (DFT) [23]. In the second study, a more accurate hybrid QM/MM (ONIOM) method was applied to study these mechanisms by including the whole protein in models [49].

**Figure 4:**
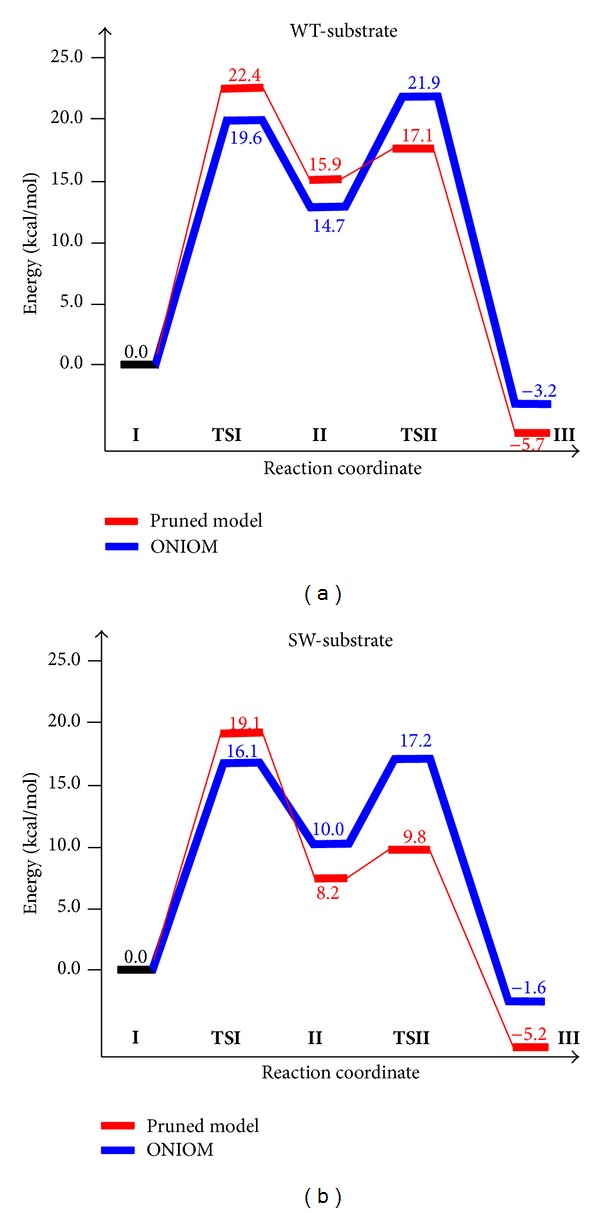
Computed barriers for the WT- and SW-substrate using pruned models (with the DFT method) and entire protein (with the ONIOM (QM/MM) method).

According to the DFT study, the formation of the gem-diol intermediate in the first step is the rate-determining step of the entire mechanism for both substrates. The reaction proceeds through a barrier of 22.4 kcal/mol and 19.1 kcal/mol (**TSI**) for the WT- and SW-substrate, respectively. The gem-diol intermediate (**II**) for the SW-substrate was found to be 8.0 kcal/mol lower in energy than the one for the WT-substrate. The stabilization of this intermediate is also reflected during the cleavage of the peptide bond through transition state** TSII. **The cleavage of the peptide bond proceeds through a barrier of 17.1 and 9.8 kcal/mol for the WT- and SW-substrates, respectively. The potential energy surface (PES) diagrams for these reactions are shown in Figure 4. The structural differences in the microenvironment of the active site play a critical role in lowering the barrier for the SW-substrate. Although the pruned models accurately reproduced the experimentally observed barrier c.a. 18.0 kcal/mol for both substrates [50], contribution of the steric and electrostatic effects of the protein surrounding on the computed energetics of the reaction was completely ignored. Therefore, to incorporate the effect of the protein environment on the energetics, the hybrid QM/MM (ONIOM:B3LYP/Amber) method was applied to investigate the hydrolytic cleavage of the WT- and SW-substrate. The PES diagrams for these reactions are shown in Figure 4. In this case, the formation of the gem-diol intermediate occurs with barriers of 19.6 and 16.1 kcal/mol for the WT- and SW-substrates, respectively. The inclusion of the electrostatic and steric effects of the surrounding protein in the model reduced the barrier of this step by ~3.0 kcal/mol for both substrates. These reductions are observed due to the alterations in the reaction coordinates and microenvironment of the active site and are not due to the long range structural reorganization in the enzyme. The creation of the gem-diol intermediate in this step for the SW-substrate (10.0 kcal/mol) is 4.7 kcal/mol more favorable than for the WT-substrate (14.7 kcal/mol). The cleavage of the peptide bond of the WT-substrate occurs through a calculated barrier of 21.9 kcal/mol. For the SW-substrate, the barrier for this process is reduced by 4.7 kcal/mol, that is, 17.2 kcal/mol from the reactant. Results from the DFT and QM/MM studies indicate that the formation of the gem-diol intermediate is the rate determining step of the entire mechanism for both substrates. The recent crystallographic [51] and Car-Parrinello MD simulations [20] studies on another member of the aspartyl protease family, HIV protease, also suggested the cleavage of the peptide bond to be the rate-limiting step. In our QM/MM calculations, the rate-limiting step for the SW-substrate is hard to distinguish due to a small difference (1.1 kcal/mol) in barriers between the two steps. The overall reduction in the barrier for each step in the SW-substrate is also in line with the experimental observation [50] suggesting that BACE1 can cleave the SW-substrate more efficiently than the WT-substrate. These studies explicitly provided an atomic level understanding of the complex mechanism of peptide bond cleavage by BACE1.

## 6. Effect of Protonation State of the Catalytic Asp Dyad in Ligand Design

The design of potent drug like molecules for the inhibition of BACE1 is an intensive area of research. However, scientific efforts to develop BACE1 inhibitors with appropriate pharmacokinetic and pharmacodynamics properties have not been successful [52, 53]. In search of novel inhibitors, a number of peptidic and nonpeptidic small molecules were cocrystallized with the ectodomain of BACE1 [52]. Important information such as ligand-protein interaction network and conformation of the ligand at the active site can be obtained from cocrystal structures. But a deeper understanding of microenvironments such as the protonation states of the Asps or the ionizable groups is still beyond the scope of X-ray crystallography. This information is absolutely necessary for the design of novel, specific inhibitors that can cross the blood brain barrier (BBB). Therefore, current BACE1 inhibitor design efforts intensively focused on the determination of the protonation states of these two Asp residues (Asp32 and Asp228) in the presence of a specific functional inhibitor scaffold [26, 48, 54–57].

**Figure 5:**
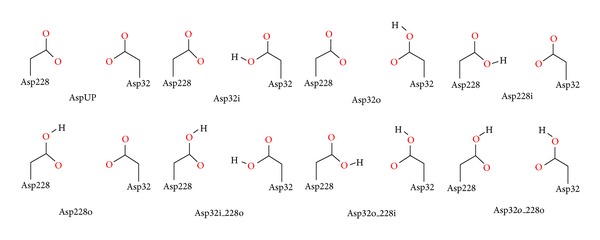
Possible protonation states of the two catalytic Asp residues.

To address the protonation states of the Asp dyad in the presence of different scaffolds of inhibitors, a molecular docking study was conducted including the critical Asp dyad (Asp32 and Asp228) of BACE1 using eight chemically diverse inhibitors [58]. In total, eight structurally different scaffolds (HE, AE, TC, CA, RA, AK, ABP, and AP (Table 1)) [43, 56, 59–64] containing inhibitors were self-docked into the corresponding X-ray structures, using the two different docking engines AutoDock 4.0 [65, 66] and Glide XP [67, 68]. In these procedures, eight possible protonation states of the catalytic Asps (AspUP, Asp32i, Asp32o, Asp228i, Asp228o, Asp32i_228o, Asp32o_228i, and Asp32o_228o) were considered (Figure 5). The results obtained from these docking simulations were analyzed using the following three parameters: (i) rms deviation of the docked ligand pose from the crystallographic orientation of the ligand that should be below 2 Å, (ii) ligand docking score: the more negative the score is, the more favorable the interaction with BACE1 is, and (iii) minimum deviation of the critical atoms that interact with the Asp dyad of the docked pose from the X-ray structure.

**Figure 6:**
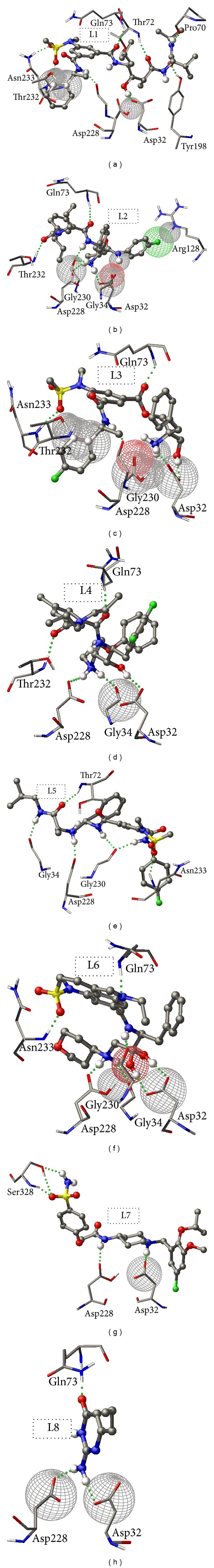
Most favorable binding modes of the ligands L1–L8 ((a) to (h), resp.) obtained from the docking procedure.

The possible protonation states were considered only if these three criterions were satisfied. The self-docking study clearly showed that the binding mode of these inhibitors depends on the protonation states of the two Asp residues. The monoprotonated Asp228i state was found to be preferred by HE (L1), TC (L3), AK (L6), and ABP (L7) based inhibitors, whereas RA (L5) prefers the monoprotonated Asp32o state. The AE (L2), CA (L4), and AP (L8) inhibitors favored the dideprotonated AspUP state. The most favorable docking poses are shown in Figure 6. Although some discrepancies were observed in predicting the protonation state of the L3 ligand, the predicted docked scores showed a good correlation (*R* ^2^ value of > 0.75) between the calculated binding affinities and experimental data. Moreover, the results obtained from this study also showed that, besides favoring a single protonation state, multiple protonation states could also be favorable for a single ligand. It is interesting to note that five out of eight ligands (L1, L2, L3, L4, and L5) showed high preference for a diprotonated state. The possibilities of multiple protonation states also agree with a previous study that suggested that the protonation states of the Asp dyad can alter under different pH conditions in the presence of the diverse inhibitors [69]. The results obtained from this study clearly indicate that considering a single protonation state of these two critical Asp residues is not sufficient and the most favored states for definite chemical scaffold must be determined prior to conducting a virtual screening campaign.

## 7. Summary and Future Perspective

This review summarizes the application of computational methodologies to elucidate the dynamics, substrate specificity, and catalytic mechanism of one of the most critical enzymes (BACE1) involved in the pathogenesis of AD. In addition, the effects of protonation states of the catalytic Asp residues have been discussed in developing novel inhibitors against this enzyme. Although the results obtained from these studies support experimental observations, the accuracy of the structural information concerning the enzyme-substrate interactions requires additional advancement. In this aspect, the application of more sophisticated computational approaches such as meta-, accelerated, dissipative particle, and polarizable dynamics simulations will be very useful. In addition to substrate-protein interaction, the studies of catalytic mechanisms can be further improved by employing more sampling methods and incorporating the hydrophobic interactions. Therefore, coupling of QM/MM method with MD simulations and incorporation of the dispersion term in the DFT methods will provide more accurate energetics. Finally, for the prediction of the protonation state of the catalytic Asp, more accurate free energy based calculations such as application of the orthogonal space random walk (OSRW) method or constant pH simulation methods will be very useful.

## Figures and Tables

**Table 1:** Ligands with chemical structure and PDB ID of cocrystal structure of BACE1.